# Sperm oxidative damage acquired during seminal plasma removal for assisted reproductive technology is reduced by BGP-15

**DOI:** 10.1007/s10815-025-03418-4

**Published:** 2025-02-11

**Authors:** Macarena B. Gonzalez, Ryan D. Rose, Haley S. Connaughton, Gracie Mackintosh, Caitlyn Bugeja, Michael Barry, Nicole O. McPherson, Rebecca L. Robker

**Affiliations:** 1 Robinson Research Institute, School of Biomedicine, The University of Adelaide, Adelaide, SA 5005 Australia; 2 Genea Fertility SA, St. Andrews Hospital, South Terrace, Adelaide, SA 5000 Australia; 3 Freemasons Centre for Male Health and Wellbeing, The University of Adelaide, Adelaide, SA 5005 Australia

**Keywords:** DNA damage, Male, Reproduction, Semen analysis, Spermatozoa*

## Abstract

**Purpose:**

Semen manipulation for assisted reproductive technology (ART) causes spermatozoa damage; thus, we investigated the potential of the novel therapeutic BGP-15 to preserve sperm quality during semen washing prior to insemination.

**Methods:**

Donated human ejaculates (*N* = 40), with or without 10 µM BGP-15, were analyzed for sperm motility, DNA fragmentation, and oxidation. Seminal plasma was removed using different clinical sperm selection methods: simple wash, swim-up, or density gradient centrifugation (DGC), followed by assessment for sperm motility, mitochondrial ROS (mtROS), mitochondrial membrane potential (MMP), and DNA fragmentation and oxidation.

**Results:**

Donated semen samples incubated with BGP-15 had increased sperm motility (+ 15%, *p* = 0.002) and reduced oxidative DNA damage levels (− 57%, *p* = 0.03).

Samples processed by simple wash had the highest sperm count compared with DGC (+ 55%, *p* < 0.005) and swim-up (+ 21%, *p* < 0.0005). Swim-up showed increased vitality compared with DGC (+ 18%, *p* < 0.001) and simple wash (+ 27%, *p* < 0.0001), as well as the lowest DNA oxidation levels compared with simple wash − 40%, (*p* = 0.01) and DGC (− 76%, *p* < 0.0001). Swim-up also had the lowest mitochondrial membrane potential compared with simple wash and DGC (− 28%, *p* < 0.03).

Comparison between untreated and BGP-15-treated groups for each sperm washing method showed that BGP-15 increased MMP in DGC sperm (+ 11%, *p* = 0.0006), and reduced DNA fragmentation in washed samples (− 22%, *p* = 0.03). Moreover, BGP-15 lowered DNA oxidation in all preparation methods: washed (− 48%, *p* = 0.002), swim-up (− 42%, *p* = 0.04), and DGC (− 29%, *p* < 0.0001).

**Conclusions:**

The inclusion of BGP-15 during semen preparation can protect sperm quality and, in the future, may be used clinically to improve sperm selection methods.

**Supplementary Information:**

The online version contains supplementary material available at 10.1007/s10815-025-03418-4.

## Introduction

An estimated three million assisted reproduction technology (ART) cycles are performed annually worldwide; yet, embryo development to blastocyst and implantation rates are low, with only 36% cumulative delivery rate after embryo transfer [1]. Male factors are responsible for about half of all infertility cases [2], and although there are many factors influencing ART outcomes, damage to sperm DNA integrity is a major contributor [3, 4]. Use of sperm with high levels of DNA fragmentation results in lower fertilization rates after IVF insemination and, irrespective of fertilization method, is associated with reduced embryo viability [5] and may contribute to an increased risk of pregnancy loss after ART [6].

The clinical manipulation of spermatozoa for use in ART can cause significant cellular damage (reviewed in [7]), which starts as soon as semen is deposited and continues through the various washes required prior to insemination. Semen selection procedures vary between clinics and may change based on the patient’s diagnosis and treatment, but it is known that time, temperature, and selection method are important factors contributing to sperm damage during clinical manipulation. Specifically, the prolonged exposure of sperm to seminal plasma increases iatrogenic damage to sperm, resulting in increased DNA fragmentation with time [8]. Additionally, standard sperm selection methods usually involve centrifugation steps that have been shown to increase the production of mitochondrial reactive oxygen species (mtROS) [9], resulting in oxidative stress that compromises sperm motility and DNA integrity [10–12]. There is a need to better understand the impact of these techniques on sperm quality, since it would potentially affect patients’ ART success.

BGP-15, a hydroximic acid niacin-derivative [(O-[3-piperidino-2-hydroxy-1-propyl]-nicotinic amidoxime)] with mitochondrial activating properties [13, 14], is an agent that we have previously used to ameliorate cryopreservation-induced damage in human semen samples [15]. In this study, we sought to assess the damage caused to human sperm during clinical manipulation prior to ART and to explore whether the incorporation of BGP-15 during these processes can protect sperm quality.

## Methodology

### Materials

Unless indicated otherwise, reagents are from Sigma Aldrich (Sigma Chemical Co., St. Louis, MO, USA).

BGP-15 ([(O-[3-piperidino-2-hydroxy-1-propyl]-nicotinic amidoxime)], CAS 66611–37-8) was from Hangzhou Molcore Biopharmatech Co. Ltd.

### Ejaculate sample collection

Human biological material (semen) was obtained in compliance with the National Statement on Ethical Conduct in Human Research (NHMRC, 2007 and incorporating all updates). All study procedures were approved by the St. Andrew’s Hospital Human Research Ethics Committee (HREC) (Project 93) and the University of Adelaide’s HREC (33,594).

Human semen samples that were discarded as excess to patient treatment were provided by collaborative partners at Genea Fertility SA, St. Andrews Hospital (Adelaide, SA) following informed consent from patients. Semen samples were produced by masturbation after at least 48 h abstinence and collected into 50 mL, sterile polypropylene sample containers (Sarstedt AG & Co. KG, Nümbrecht, Germany). Samples were excluded from analysis if total sperm count was < 5 million spermatozoa, seminal volume was < 1 mL, or total time between specimen deposit and arrival to the research laboratory was > 4.0 h.

### Simulated semen manipulation and sperm selection prior to ART

**Fig. 1:**
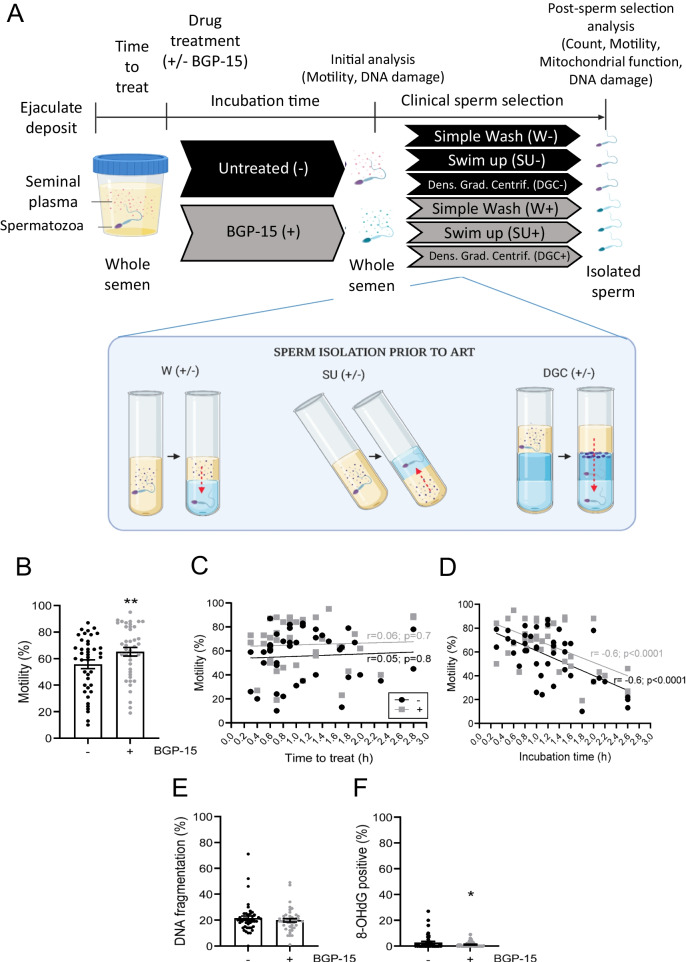
BGP-15 maintains sperm motility and reduces DNA oxidation in semen samples. **A** Experimental design simulating clinical waiting times and sperm selection methods prior to ART. Patient semen specimens were deposited and waited for use in IVF/ICSI (time to treat). Discarded semen was split, with gamete media containing or lacking BGP-15 added 1:1 v/v (Incubation time). Initial analyses (motility, DNA fragmentation, and DNA oxidation) were performed on untreated ( −) and BGP-15-treated ( +) samples. Each aliquot was then processed using one of three sperm selection methods: wash (W; sperm cells are separated from seminal plasma components), swim-up (SU; highly motile sperm swim into an overlying gamete media layer), or density gradient centrifugation (DGC; motile, viable sperm are concentrated in the pellet while less dense components remain in upper layers), with or without BGP-15. Selected sperm were assessed for count, motility, viability, membrane fluidity, mitochondrial activity (MMP, mtROS), DNA fragmentation, and DNA oxidation. Analyses in panels **B–F** refer to the initial analysis of the untreated ( −) and BGP-15-treated ( +) whole semen samples prior to sperm selection. **B** Sperm motility measured as the percentage of total motile sperm cells. **C** Relationship between time to treat the sample (time elapsed from ejaculate deposit until BGP-15 treatment) and sperm motility at initial semen analysis. **D** Relationship between incubation time (total time the ejaculate sample was exposed to BGP-15) and sperm motility at initial analysis. **E** Sperm DNA fragmentation measured as the percentage of sperm negative for HALO/SCD assay. **F** Sperm DNA oxidation measured as the percentage of sperm positive for nuclear 8OHdG immunodetection. *N* = 40 ejaculates. Data shown as mean ± SEM. Statistical analysis was either paired, **B, E, F** two-tailed Student’s *t*-test or **C, D** linear regression analysis. *p < 0.05 and **p < 0.01

To investigate the protective effect of BGP-15 in human sperm, we designed an experimental protocol that simulated clinical manipulation of sperm and assessed damage to sperm quality due to time (clinical waiting times and transport to the research laboratory) and physical factors (sperm selection methods) (Fig. 1A). Firstly, the semen sample is deposited by the patient and sits at room temperature (RT) at the clinical laboratory waiting to be used for ART (Time to treat). Once a 1 mL aliquot has been taken for use in IVF/ICSI, the excess whole semen is split in half and each aliquot is mixed 1:1 v/v with gamete medium (Vitrolife Pty Ltd., Sydney, Australia), either containing or not BGP-15 at a final concentration of 10 µM BGP-15 per sample. Both untreated and BGP-15-treated specimens are then delivered by courier at RT to the research laboratory at the University of Adelaide and assessed for sperm motility, DNA fragmentation and oxidation.

After this, each sample is then split again into three aliquots per group that undergo different sperm selection processes: simple wash, swim-up, and density gradient centrifugation (described in detail below), following the recommended protocols from the World Health Organization (WHO) 5th Edition Criteria Guidelines [16]. Briefly:

- Simple wash or wash (W): Semen volume is made up to 3 mL with Biggers, Whitten and Whittingham (BWW, [17]) media containing 1 mg/ml polyvinyl alcohol (PVA), then centrifuged for 20 min at 500 × *g*. Supernatant is removed; then the cell pellet is resuspended in BWW medium and centrifuged again for 10 min at 500 × g. Supernatant is removed again, and cell pellet resuspend in 1 mL of BWW medium.
- Swim-up (SU): 1.5 mL of BWW medium is underlaid with 0.5–1.0 mL of the semen specimen. The tube is then placed on a 45° angle for 45 min at 37 °C, with the uppermost 1 mL of media collected after this time.
- Density gradient centrifugation (DGC): SpermGrad stock solution (considered “100%”; Vitrolife) is diluted to 80% and 40% with BWW medium. 1.5 mL of 40% solution is overlaid with 1.5 mL of 80% solution, forming an interphase between the two. Then the semen is overlaid on top, and subsequently centrifuged for 20 min at 500 × *g*. The cell pellet is isolated and resuspended in BWW medium, then centrifuged again for 10 min at 500 × *g*. Supernatant is removed and the cell pellet is resuspended in 1 mL of BWW medium.

Depending on the type of sperm selection method, ejaculate sample aliquots treated with BGP-15 were processed and/or resuspended using BWW or SpermGrad media (as appropriate) containing 10 µM BGP-15 as well.

After sperm selection, a detailed sperm quality analysis is performed, including sperm count, motility, viability, membrane fluidity, mitochondrial reactive oxygen species (mtROS), mitochondrial membrane potential (MMP), DNA fragmentation, and DNA oxidation, as detailed below.

### Semen analysis

Sample volume, sperm count, viability, and motility are assessed following the World Health Organization 5th edition criteria guidelines [16]. Briefly, semen is diluted 1:20 in sperm diluting fluid (5% w/v sodium bicarbonate, 1% w/v formaldehyde in MilliQ water); then 10µL is used to count the number of sperm cells per mL using a hemocytometer. Sperm motility is assessed in 200 cells per sample and expressed as a percent from total sperm using light microscope. Sperm viability is assessed by Live/Dead Far Red vitality stain using flow cytometry (described below) and then expressed as percent negative. All samples are assessed blinded to the treatment group.

### Sperm membrane fluidity, mitochondrial function, and cell viability assays

To measure membrane fluidity, cells were stained with 0.27 μM Merocyanine 540 (M540) (Calbiochem, Burlington, MA, USA) and with Live/Dead Fixable Far Red dead cell stain kit (Life Technologies, Waltham, MA, USA) for 30 min. To detect mtROS, cells were stained with 2 μM MitoSOX Red (MSR) (Life Technologies) and 0.05 μM Sytox Green (Life Technologies) for 15 min. To assess MMP, cells were stained with 2 μM JC-1 (Life Technologies) and with Live/Dead Fixable Far Red dead cell stain kit (Life Technologies) for 15 min.

For all assays, cells were incubated in the dye solution at 37 °C, shielded from light. This was followed by centrifugation at 500 × *g* for 5 min and resuspension in BWW/PVA. Analysis was conducted on a FACS-Canto II Flow cytometer (Becton Dickinson, CA). Emission measurements were made using 530/30 band-pass (green), 585/42 band-pass (red), and > 670 long-pass (far red). Forward scatter and side scatter measurements were taken to generate a scatter plot, which was used to gate for sperm cells only, excluding any larger contaminating cells. A total of 10,000 events were recorded for each sample. Data was analyzed using FlowJo LLC (BD Biosciences, Becton Dickinson).

### Sperm DNA integrity assays

DNA fragmentation was assessed by the sperm chromatin dispersion (SCD) test, also known as “Halo” test. Cells were thawed and mixed with 1% low melting agarose to a final concentration of 0.7% agarose at 37 °C. The cell-agarose mixture was put onto a slide pre-coated with 0.65% standard agarose. Slides were solidified at 4 °C for 4 min. Coverslips were carefully removed, and slides placed horizontally in acid denaturation solution (0.08N HCL) for 7 min at room temperature. Slides were then incubated in a neutralizing and lysing solution 1 (0.4 M Tris, 0.8 M DTT, 1% SDS, and 50 mM EDTA, pH 7) for 10 min at room temperature, then submerged into neutralizing and lysing solution 2 (0.4 M Tris, 2 M NaCl, and 1% SDS, pH 7.5) for 5 min at room temperature. Slides were then washed in Tris–borate-EDTA buffer (0.09 M Tris–borate and 0.002 M EDTA, pH 7.5) for 2 min, dehydrated in 70%, 90%, and 100% ethanol for 2 min each at room temperature and air dried. Slides were stained with DAPI (1/2000) for 10 min at room temperature. One hundred cells were counted under a fluorescent microscope for “halo” (intact, dispersed DNA) or “no halo” (fragmented, condensed DNA). Results are expressed as a percentage of Halo negative (fragmented) cells.

8OHdG immunostaining was used for detection of oxidized DNA. Cells were centrifuged at 500 g and resuspended in decondensation buffer (2 mM DL-dithiothreitol and 0.5% Triton X-100 in PBS) for 10 min at room temperature. Cells were fixed in 4% paraformaldehyde for 15 min at 4 °C, then antigen blocked using 1.5% goat serum/PBS for 1 h at room temperature and incubated after with DNA/RNA Damage Antibody (Novus Biologicals) (Littleton, CO, USA) overnight at 4 °C. Secondary antibody used was Alexa Fluor 488 Goat anti-Mouse IgG (Life Technologies). One hundred cells were counted under a fluorescent microscope at 488 nm as positive or not positive for 8OHdG staining. Results are expressed as a percentage of 8OHdG-positive cells.

### Statistical analysis and figure preparation

Data was assessed for normality using the Shapiro–Wilk and the D’Agostino-Pearson omnibus normality tests prior to analysis.

To measure the effect of BGP-15 treatment on sperm quality during clinical waiting times, a paired *t*-test was used to analyze matching data pairs for each patient. To examine the association between two sperm parameters, a linear regression test was used.

To determine the interaction of sperm selection method and BGP-15 exposure during selection, a repeated measures linear mixed model was used, with post hoc pairwise comparisons using paired Student’s *t*-test between untreated and BGP-15-treated matching data for each selection method.

Statistics software used were GraphPad Prism v008 (GraphPad Software, La Jolla, CA, USA), and IBM SPSS Statistics v28.0.1.0 (SPSS IBM, Chicago, IL, USA), for Windows. *p*-value ≤ 0.05 was considered statistically significant.

Graphs were generated using GraphPad Prism v008. Schematics were created using Biorender (Toronto, Ontario, Canada).

## Results

### BGP-15 maintains sperm motility in semen samples

Standard clinical practice is to remove seminal plasma from semen samples prior to insemination, yet the techniques used are varied. Thus, we aimed to assess and compare damage to patients’ sperm caused by the manipulation of sperm samples in a clinical setting and the ability of BGP-15 to protect sperm quality during these processes. Briefly, samples were split in half and untreated ( −) or treated ( +) 1:1 v/v with gamete medium containing BGP-15. Semen samples then waited in the BGP-15 solution until processing. At this time, an initial semen analysis was performed, and each group was then further split into three of the most commonly used clinical sperm selection techniques, namely, simple wash (W), swim-up (SU), and density gradient centrifugation (DGC), followed by a comprehensive semen analysis in the isolated sperm samples (Fig. 1A). We collected ejaculates that were excess to the ART procedure from 37 male participants (with a total of 40 semen samples collected) at a local fertility clinic. As detailed in Table 1, the average age of these men was 38 years, while the average BMI was 27.3 kg/m^2^. Baseline semen analysis results were a mean ejaculate volume of 3.7 mL, sperm count of 60.2 × 10^6^/mL, motility 52.7%, and normal morphology 7.1%.

**Table 1:** Data are presented as mean ± standard deviation (SD) or as indicated

**Number of ejaculate samples, *n***	40
**Number of male participants, *n***	37
Age (y), mean ± SD (Min–Max)	38.0 ± 5.8 (27.5–48.7)
BMI (kg/m^2^), mean ± SD (Min–Max)	27.3 ± 3.6 (20.3–34.4)
Smoking, *n* (%)	7 (18.9)
Cigarettes per day, mean ± SD (Min–Max)	10 ± 7.1 (0.5–40)
Alcohol, *n* (%)	33 (89.2)
Standard drinks per week, mean ± SD (Min–Max)	9.0 ± 9.7 (0.5–40)
Indications for treatment	
Male factor, *n* (%)	7 (18.9)
Tubal disease, *n* (%)	8 (21.6)
Endometriosis, *n* (%)	12 (32.4)
PCOS, *n* (%)	8 (21.6)
Unexplained, *n* (%)	27 (72.8)
Other, *n* (%)	29 (78.4)
**Baseline semen analysis**	
Volume, mean ± SD (Min–Max) mL	3.7 ± 1.6 (1.5–8)
Count, mean ± SD (Min–Max) × 106/mL	60.2 ± 50.5 (19.0–322.0)
Motility, mean ± SD (Min–Max) %	52.7 ± 12.8 (23.0–75.0)
Normal morphology, mean ± SD (Min–Max) %	7.1 ± 4.5 (1.0–22.0)

At the initial semen analysis, whole semen samples treated with BGP-15 had 14.6% increased motility, compared with untreated aliquots from the same ejaculate (*p* = 0.002, Fig. 1B). To investigate whether incubation time influenced the effectiveness of BGP-15 treatment in increasing sperm motility in semen samples, we conducted a regression analysis of sperm motility outcomes against (a) time to treat, defined as the time elapsed from sample deposit to the start of BGP-15 treatment (Fig. 1C) and (b) incubation time, defined as time elapsed from start of BGP-15 treatment to initial semen analysis (Fig. 1D). The majority of the samples (57.5%; 23/40) had a time to treat waiting period of 1 h, which was within the standard clinical timeframe. Almost half of the samples (45%; 18/40) had an incubation time of 1 h. Sperm motility was not significantly affected by the time to treat duration in either group (Fig. 1C), but it decreased with increasing incubation time (*p* < 0.0001, Fig. 1D). Despite this decline, the aliquot treated with BPG-15 had higher motility compared with the untreated aliquot from the same ejaculate, independent of the duration of the time to treat (*p* = 0.04, Fig. 1C) and incubation periods (*p* = 0.01, Fig. 1D).

While DNA fragmentation levels were similar between untreated and BGP-15-treated aliquots (Fig. 1E), BGP-15 treatment of whole semen reduced sperm DNA oxidation levels by 57.4% (*p* = 0.03, Fig. 1F).

These results show that diluting whole semen specimens in gamete medium containing BGP-15 promotes higher sperm motility and lower DNA oxidation, and this is not limited by strict time requirements for treatment commencement after specimen deposit or treatment incubation time.

### Sperm selection method affects sperm quality parameters

The chosen standard sperm selection methods produced the expected effects on the total number of sperm cells retrieved after processing, with wash resulting in a higher concentration (20.9 × 10^6^) than density gradient centrifugation (8.3 × 10^6^) and swim-up (3.6 × 10^6^), with similar numbers observed in the corresponding BGP-15 groups.

**Fig. 2:**
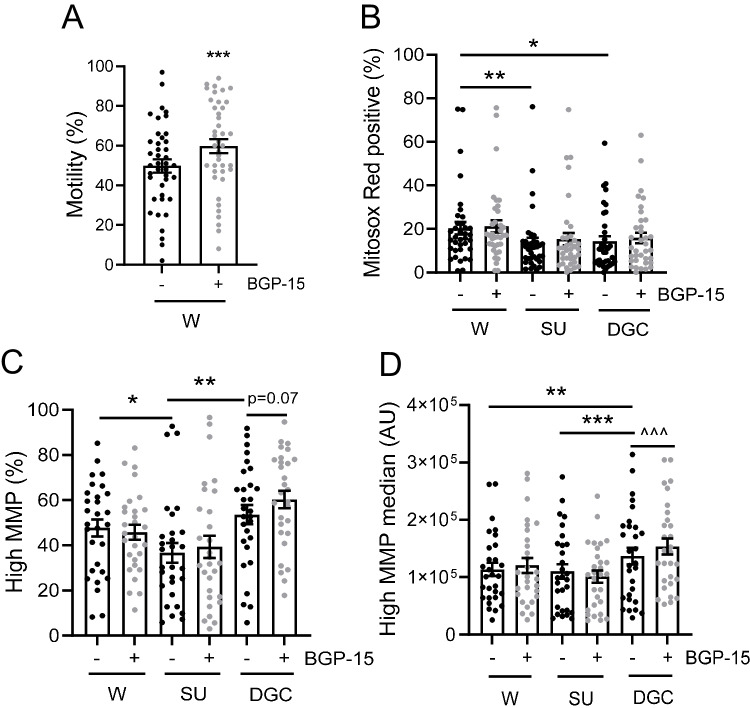
BGP-15 improves sperm motility after wash and increases sperm MMP after DGC. Semen specimens were processed by one of three methods: wash (W), swim-up (SU) and density gradient centrifugation (DGC); in the absence ( −) or presence ( +) of BGP-15. **A** Sperm motility measured as percentage of motile sperm in washed (W) samples. **B** Sperm mtROS measured as the percentage of sperm cells positive for MitoSox Red superoxide stain using flow cytometry. **C** Subpopulation of sperm displaying high MMP, measured as the percentage of sperm cells positive for red fluorescence after JC-1 mitochondrial stain using flow cytometry. **D** Fluorescence intensity in the high MMP sperm subset, measured as median arbitrary units (AU). **A, B** *N* = 38 or **C, D** *N* = 30 isolated sperm samples. Data shown as mean ± SEM. Statistical analysis was either paired, **A** two-tailed Student’s *t*-test or **B–D** repeated measures mixed model. Post hoc pairwise comparisons were paired Student’s *t*-test, either within the same treatment group (*p = 0.05, **p < 0.01, and ***p < 0.001), or between untreated and BGP-15-treated samples (^^^*p* < 0.001) from the same selection method group

Sperm treated with BGP-15 during wash had 16.7% higher motility compared with untreated, at the post-selection analysis (*p* = 0.001, Fig. 2A). Sperm selection method, but not BGP-15 treatment, affected sperm cell viability as expected (*p* < 0.0001), with swim-up yielding a greater percentage of live sperm cells than the two centrifugation based-methods, 18% higher compared with DGC (*p* < 0.001) and 27% higher compared with wash (*p* < 0.0001) (Supp Fig. 1A). There was no effect of selection method or BGP-15 treatment on membrane fluidity as detected by the Merocyanin 540 assay (Supp Fig. S1B).

Sperm selection method affected sperm mtROS levels (*p* = 0.01), with washed sperm showing mtROS levels that were on average a third higher than those from swim-up (*p* = 0.001) and density gradient centrifugation (*p* = 0.01; Fig. 2B). Assessment of sperm MMP by JC-1 showed that the selection method affected the proportion of high MMP sperm (*p* = 0.0006), with swim-up resulting in 23.1% fewer high MMP cells than wash (*p* = 0.01) and 31.5% fewer than density gradient centrifugation (*p* = 0.001). BGP-15-treated density gradient centrifugation samples tended to have more high MMP sperm cells when compared with matched untreated samples (*p* = 0.07, Fig. 2C). Similarly, both selection method and BGP-15 treatment affected MMP fluorescence intensity (*p* = 0.03), as measured by JC-1 assay. Density gradient centrifugation had on average 18.3% higher MMP fluorescence intensity than both washed (*p* = 0.02) and swim-up (*p* = 0.001) samples. Density gradient centrifugation sperm incubated with BGP-15 had 10.1% higher MMP fluorescence intensity compared with untreated from the same ejaculate (*p* = 0.0006, Fig. 2D).

These results suggest that the choice of sperm selection method can impact some sperm quality parameters, such as recovery rate, viability, and mitochondrial activity, and that BGP-15 promotes higher MMP in sperm collected through density gradient centrifugation.

### BGP-15 protects sperm DNA from oxidation during clinical selection

**Fig. 3:**
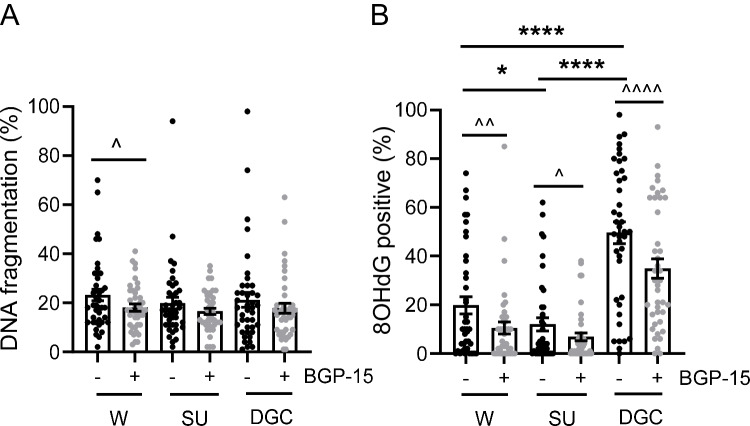
BGP-15 prevents sperm DNA oxidation during the clinical selection of sperm. Semen specimens were processed by wash (W), swim-up (SU), and density gradient centrifugation (DGC); in the absence ( −) or presence ( +) of BGP-15. **A** Sperm DNA fragmentation levels measured as the percentage of sperm cells that were negative for HALO/SCD assay. **B** Sperm DNA oxidation levels measured as the percentage of sperm positive for nuclear 8OHdG immunodetection. *N* = 38 isolated sperm samples. Data shown as mean ± SEM. Statistical analysis was repeated measures mixed model. Post hoc pairwise comparisons were paired Student’s *t*-test, either between untreated groups only (*p = 0.05, ****p < 0.0001), or between untreated and BGP-15-treated samples from the same method group (^*p* < 0.05, ^^*p* < 0.01, and ^^^^*p* < 0.0001)

While there was no effect of selection method on sperm DNA fragmentation levels, there was an overall reduction of sperm DNA fragmentation levels in BGP-15-treated samples (*p* = 0.02), with a significant 22% reduction in washed samples (*p* = 0.03, Fig. 3A). Sperm DNA oxidation levels were affected by both selection method and BGP-15 exposure (*p* = 0.04), with density gradient centrifugation producing the highest levels of sperm DNA oxidative damage (49.6%), followed by wash (19.8%), then swim-up (12%). BGP-15 treatment reduced sperm DNA oxidation levels by 47% in wash (*p* = 0.002), 43% in swim-up (*p* = 0.04), and 30% in density gradient centrifugation (*p* < 0.0001) (Fig. 3B). These results combined indicate that sperm selection processes induce sperm DNA fragmentation and oxidation but that the addition of BGP-15 can partially alleviate this damage.

## ,Discussion

This study highlights the potential of BGP-15 as a protective agent in clinical sperm preparation, demonstrating its ability to preserve sperm motility, maintain mitochondrial function, and reduce DNA oxidation across various selection methods. These findings are particularly significant given the routine use of sperm selection in ART, which can inadvertently cause oxidative damage and compromise sperm integrity.

Our results revealed that BGP-15 consistently improved sperm motility, regardless of the time elapsed between sample collection and processing. This constitutes an advantage, as clinical settings often encounter logistical delays, or in the case of procedures like testicular sperm aspiration (TESA) or testicular sperm extraction (TESE), where the sperm is suspended in medium during collection [18]. The observed motility enhancement suggests that BGP-15 supplementation could mitigate the decline in sperm quality associated with prolonged handling times, potentially bridging gaps in clinical workflows.

Sperm selection methods influenced multiple parameters of sperm quality, with the expected different impacts on viability, mitochondrial function, and DNA integrity [19–21]. Swim-up maintained the highest sperm vitality and lowest mtROS levels but did not outperform centrifugation-based methods in reducing DNA fragmentation. Notably, density gradient centrifugation (DGC) resulted in the highest mitochondrial membrane potential (MMP), correlating with enhanced mitochondrial activity [22], a marker of sperm competence [23]. However, this benefit was offset by increased oxidative damage. Some male infertility diagnosis [21, 24] or lifestyle factors [25] can affect sperm DNA integrity and make it more susceptible to damage under post-ejaculatory stresses like sperm manipulation and selection. Due to this and the fact that there are clinics where swim-up is used in combination with DGC [26], more attention should be paid to each individual patient’s sperm sensitivity to damage during clinical processes, since there is some evidence it can lead to fertilization failure after IVF [27]. To avoid this, a trial semen preparation prior to the egg collection cycle should be performed to choose the most appropriate technique, as advised by good IVF practice guidelines [28].

The inclusion of BGP-15 in sperm selection media had a marked protective effect against sperm DNA oxidation across all methods. This is especially important as sperm DNA oxidation has become recognized in recent years as a marker of sperm quality [29] and a predictor of ART success [30]. While the exact molecular mechanism of action for BGP-15 in sperm is not fully understood, its established role in modulating mtROS production [31–33] provides a plausible explanation for its protective effects and is consistent with our previous research demonstrating that BGP-15 enhances MMP and reduces sperm mtROS and DNA oxidation levels [15, 34, 35], ultimately supporting embryo development [34, 35].

Sperm motility [36], mitochondrial function [37], and DNA integrity [30, 38] are key predictors of ART success, including fertilization, embryo quality, and live birth rates. The ability of BGP-15 to mitigate oxidative damage and maintain sperm functionality during clinical processing could enhance the efficacy of ART procedures, particularly in cases of male infertility or suboptimal sperm quality. However, our study used discarded excess ejaculates from a fertility clinic, which may not fully represent the diversity of male patients undergoing ART. Variability in sperm quality, fertility diagnoses, and underlying conditions could affect the generalizability of the results and future studies should target specific subsets of patient populations to corroborate the efficacy of BGP-15 treatment. In line with this, future research should link the improvements in sperm quality parameters by BGP-15 to clinical outcomes such as fertilization rates, embryo quality, pregnancy rates, or live birth rates to further validate its clinical utility. Further studies utilizing Computer-Assisted Sperm Analyzer (CASA) instruments to measure sperm motility more precisely and assess a greater number of motility parameters would be informative.

In summary, this study shows that BGP-15 is a promising adjunct in ART, capable of preserving critical sperm quality parameters during clinical preparation. Furthermore, the protective effects of BGP-15 may allow for greater flexibility in sperm handling protocols, reducing the pressure for stringent time constraints and enabling more patient-specific approaches. Its inclusion in sperm selection protocols could minimize the detrimental effects of oxidative stress, optimize sperm functionality, and improve ART outcomes.

## Conclusion

Our study confirms that clinical processes should become more tailored to the individual patient to potentially increase ART performance and identifies BGP-15 as a media supplement that is effective to reduce DNA damage during clinical sperm manipulation.

## Supplementary Information

Below is the link to the electronic supplementary material.

Supplementary file1 (PDF 122 KB)

## Data Availability

N/A.
