# Supplementary material for: Sperm oxidative damage acquired during seminal plasma removal for assisted reproductive technology is reduced by BGP-15
Source: J Assist Reprod Genet. 2025 Feb 11;42(4):1133–42. doi: 10.1007/s10815-025-03418-4 (PMC12055704; doi:10.1007/s10815-025-03418-4)
Supplement: Supplementary file 1 — Supplementary file1 (PDF 122 KB) [file 10815_2025_3418_MOESM1_ESM.pdf]

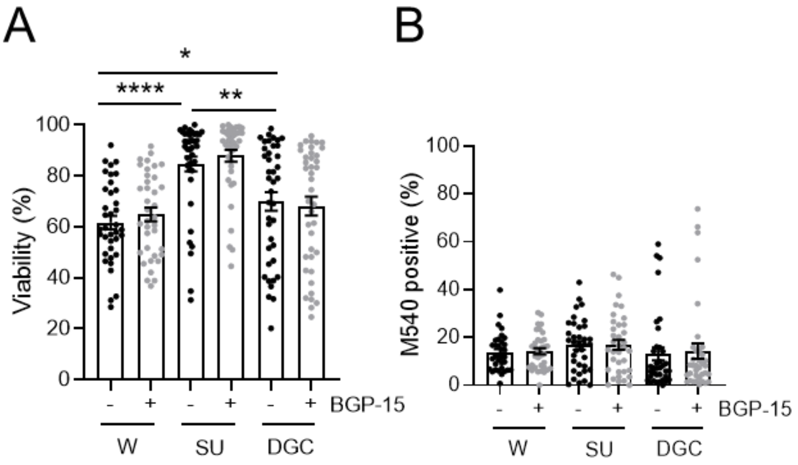

Supplementary Figure S1. Effect of clinical sperm isolation techniques on sperm.

Semen samples were purified by one of three methods: wash (W), swim-up (SU) and density gradient centrifugation (DGC); in the absence (-) or presence (+) of BGP-15. **A.** Viability was expressed as percentage of sperm cells negative for dye stain using Far Red Dead/Live stain by flow cytometry. **B.** Membrane fluidity measured as percentage of sperm positive for Merocyanin 540 (M540) membrane dye using flow cytometry. N=37 (A), or N=36 (B) isolated sperm samples. Data shown as mean ± SEM. Statistical analysis was repeated measures mixed model. Post-hoc pairwise comparisons were paired Student's T-test, either between untreated groups only (*p=0.05, **p<0.01, ****p<0.0001), or between untreated and BGP-15-treated samples from the same method group.
